# Timing Precision in Population Coding of Natural Scenes in the Early Visual System

**DOI:** 10.1371/journal.pbio.0060324

**Published:** 2008-12-16

**Authors:** Gaëlle Desbordes, Jianzhong Jin, Chong Weng, Nicholas A Lesica, Garrett B Stanley, Jose-Manuel Alonso

**Affiliations:** 1 Coulter Department of Biomedical Engineering, Georgia Institute of Technology and Emory University, Atlanta, Georgia, United States of America; 2 Department of Biological Sciences, State University of New York College of Optometry, New York, New York, United States of America; 3 Division of Neurobiology, Department Biology II, Ludwig-Maximilians-Universität, Munich, Germany; University of California Berkeley, United States of America

## Abstract

The timing of spiking activity across neurons is a fundamental aspect of the neural population code. Individual neurons in the retina, thalamus, and cortex can have very precise and repeatable responses but exhibit degraded temporal precision in response to suboptimal stimuli. To investigate the functional implications for neural populations in natural conditions, we recorded in vivo the simultaneous responses, to movies of natural scenes, of multiple thalamic neurons likely converging to a common neuronal target in primary visual cortex. We show that the response of individual neurons is less precise at lower contrast, but that spike timing precision across neurons is relatively insensitive to global changes in visual contrast. Overall, spike timing precision within and across cells is on the order of 10 ms. Since closely timed spikes are more efficient in inducing a spike in downstream cortical neurons, and since fine temporal precision is necessary to represent the more slowly varying natural environment, we argue that preserving relative spike timing at a ∼10-ms resolution is a crucial property of the neural code entering cortex.

## Author Summary

Neurons convey information about the world in the form of trains of action potentials (spikes). These trains are highly repeatable when the same stimulus is presented multiple times, and this temporal precision across repetitions can be as fine as a few milliseconds. It is usually assumed that this time scale also corresponds to the timing precision of several neighboring neurons firing in concert. However, the relative timing of spikes emitted by different neurons in a local population is not necessarily as fine as the temporal precision across repetitions within a single neuron. In the visual system of the brain, the level of contrast in the image entering the retina can affect single-neuron temporal precision, but the effects of contrast on the neural population code are unknown. Here we show that the temporal scale of the population code entering visual cortex is on the order of 10 ms and is largely insensitive to changes in visual contrast. Since closely timed spikes are more efficient in inducing a spike in downstream cortical neurons, and since fine temporal precision is necessary in representing the more slowly varying natural environment, preserving relative spike timing at a ∼10-ms resolution may be a crucial property of the neural code entering cortex.

## Abstract 3

Early neural representation of visual scenes occurs with a temporal precision on the order of 10 ms, which is precise enough to strongly drive downstream neurons in the visual pathway. Unlike individual neurons, the neural population code is largely insensitive to pronounced changes in visual contrast.

## Introduction

The precision of neuronal spike trains is at the center of a fundamental debate in neuroscience as to what aspects of neuronal signaling are important in representing information in the brain. Individual neurons can have extremely precise and repeatable responses to the visual stimuli that strongly drive them (down to 1-ms variability) [1–5], but they exhibit seemingly degraded temporal precision of firing activity in response to suboptimal stimuli [5–10]. In the presence of natural scenes, the activity of individual neurons is sparse [11] and precisely timed across repeated presentations of the visual stimulus, even though natural stimuli tend to vary on a time scale that is several times slower [12]. However, in most natural circumstances, the brain does not have access to multiple repetitions of the same identical stimulus, and, therefore, it is the precision of spiking across neuronal sub-populations on single trials that is ethologically relevant. While synchrony across neurons in the retina and visual cortex has been reported at various time scales, which can depend on the visual stimulus [10,13], the temporal precision of the neural code directly entering primary visual cortex, and its dependence on the stimulus, are still unknown. We used natural visual stimuli to investigate the spike timing precision of populations of geniculate neurons that serve as the direct input to visual cortex. We show that the response of individual neurons is less precise across stimulus repetitions when luminance contrast is reduced. However, this reduction in the precision of spike timing is not observed at the level of the neuronal population. Therefore, spike timing precision in populations of geniculate neurons is relatively insensitive to global changes in visual contrast and remains constant on the order of ∼10 ms. Since closely timed spikes from either a single neuron [14] or several neurons [15] are more likely to induce a spike in the downstream cortical neuron to which they are projecting, and since fine temporal precision is necessary in representing the more slowly varying natural environment [12], preserving the relative timing of spikes at a resolution of ∼10 ms may be a crucial aspect of the neural code entering primary visual cortex.

## Results

**Figure 1:**
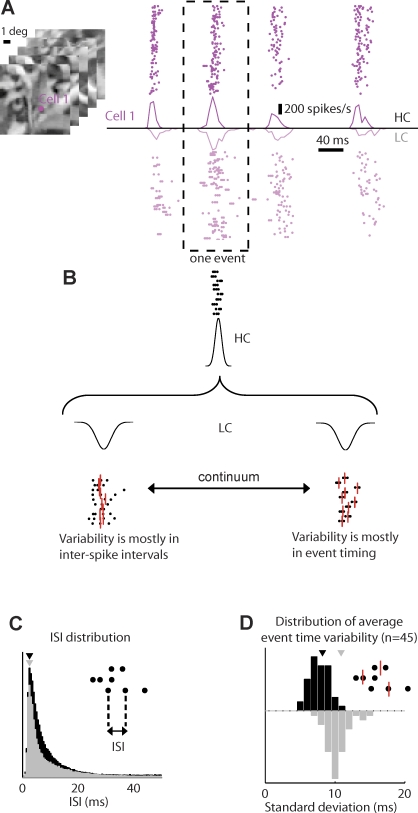
Spike Timing Precision in Single Cells (A) Movies of natural scenes were presented while recording from individual cells in the LGN. Left panel: The receptive field of a typical LGN X ON cell is superimposed, to scale, on the first frame of the natural visual stimulus. Right panel: Raster plots and PSTH for the response of this cell during a 500-ms segment of the movie, at HC and LC. (B) Possible sources of spike timing variability composing each PSTH event, where the red tick marks denote the median time of each event. (C) Distribution of ISIs (as defined in inset) over all cells (*n* = 45), at HC (black) and LC (gray). Arrows indicate distribution peaks. (D) Distribution of the variability in median event time (as defined in inset) for all events in all cells, at HC (black) and LC (gray).

A short movie of a natural scene recorded from a “cat-cam” [16] was presented repeatedly to anesthetized cats while recording extracellular activity of multiple single units in the lateral geniculate nucleus (LGN) in vivo. To test how spike timing precision in individual cells and cell populations was affected by the properties of the visual stimulus, each group of cells was stimulated with both a high-contrast (HC) version and a low-contrast (LC) version of the movie. Figure 1A shows the firing activity of a typical ON-center geniculate cell in response to a 500-ms section of the movie presented at both high and low contrast. Each line in the raster plot corresponds to a single repeat and shows the spikes generated during one presentation of this movie section. The peri-stimulus time histogram (PSTH) shows the summed response across 64 repeated presentations. The geniculate neurons exhibited a typical pattern of response in which brief intervals of silence lasting 15 ms or more alternated with firing “events” [6], or groups of closely spaced spikes lasting up to 100 ms (median: 30 ms), which consistently occurred at approximately the same time on each movie presentation. A 63% reduction of the luminance contrast in the movie resulted in a 23% reduction in the neuronal firing rate, from 10.5 spikes/s at HC to 8.1 spikes/s at LC on average across cells (see full distribution in Figure S1A), and in a latency increase of 3.4 ms (Figure S1B). The firing rate reduction and latency increase are visible as a decrease in height and a shift in time in the PSTH events at LC in the particular example of Figure 1A.

PSTH events are typically wider in duration at LC than at HC [17] (as in the example rasters and PSTHs shown in Figure 1A, where the average event full width at half-height is 7 ms at HC, 11 ms at LC). Therefore, it has been assumed that suboptimal stimulation leads to a decrease in the temporal precision of the response. However, the widening of the PSTH events from the HC to LC condition could, in principle, arise from several sources, which have different functional implications: either an increase in the inter-spike intervals (ISIs) within the event on each trial (Figure 1B, left), or an increased variability in the timing of the events themselves from trial to trial (Figure 1B, right), or some combination of both factors. Further analyses were performed to test these two possibilities. First, over all cells, the distribution of (within-trial) ISIs was very similar at HC and LC. In Figure 1C, both distributions peak at 2.5 ms and have similar full width at half-height (8 ms at HC, 6 ms at LC). If ISIs were longer at LC than HC, the ISI distribution would be wider at LC than HC, which is the opposite of what we find. The fact that the ISI distribution at LC decays faster than at HC is simply due to the higher firing rate at HC, as is the case for Poisson processes (e.g., see [3]). Second, the timing of events showed significantly more across-trial variability (or “jitter”) at LC (10.9 ± 1.6 ms mean ± standard deviation) than HC (8.2 ± 1.3 ms), with a 2.7-ms difference on average across cells (paired *t*-test, *p* < 0.01, *n* = 45 cells; see Figure 1D). These results suggest, therefore, that overall spike timing variability is primarily due to across-trial variability in the timing of the event as a whole.

**Figure 2:**
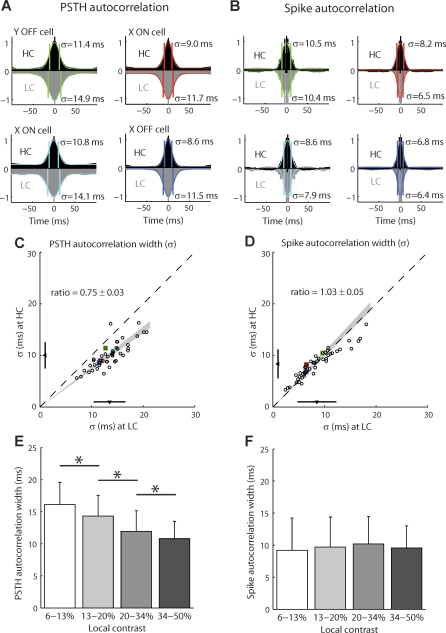
Firing Event Timing Precision Changes with Contrast, but Spike Timing within Events Does Not (A and B) Examples of PSTH autocorrelation (A) and spike autocorrelation (B) for four typical cat LGN cells. In each plot, the temporal width of the autocorrelation function is measured as the standard deviation of the Gaussian function that best fits it in the least-mean-square sense, both at HC (top half of each plot) and LC (bottom half). (C) Temporal width of PSTH autocorrelation for all cells (*n* = 45). The dashed line is the unity line. The shaded area indicates the 95% confidence estimate of the mean HC/LC ratio of the widths. The mean ± standard deviation are represented along each axis by an arrowhead and bar. The four cells illustrated in (A) and (B) are highlighted in the same colors in (C) and (D). (D) Temporal width of spike autocorrelation for all cells (*n* = 45). Same conventions as in (C). (E) Temporal width of PSTH autocorrelation for all cells at four levels of local contrast. Each bar represents the mean + 1 standard deviation (*n* = 45 cells) at the corresponding range of local contrast. Note that the error bars, which represent standard deviation, are not by themselves indicative of whether two distributions have significantly different means. Instead, we used asterisks to indicate that two means are statistically significantly different (paired *t*-test, *p* < 0.05). (F) Temporal width of spike autocorrelation for all cells (*n* = 45) at the same four levels of local contrast as in (E). Each bar represents the mean + 1 standard deviation.

Ultimately, to compare within-cell and across-cell variability, correlation analysis is necessary. Therefore, we first re-examined and validated these single-cell precision findings in terms of correlation measures. First, we quantified the average PSTH event duration by measuring the width of the PSTH autocorrelation function, in which the temporal relationship between individual spikes is lost. We also quantified the temporal precision of individual spikes in a spike train by measuring the width of the spike autocorrelation function (i.e., the autocorrelation of the full spike train). As detailed in Figure S2, the width of the PSTH autocorrelation includes two possible sources of spike timing variability (within-trial and across-trial), whereas the temporal width of the spike autocorrelation function corresponds only to the within-trial variability. The PSTH autocorrelation and spike autocorrelation functions are shown in Figure 2A and 2B for four typical LGN cells at HC (top) and LC (bottom), with the temporal widths in each condition indicated. The PSTH autocorrelation functions were significantly wider at LC than at HC on average (Figure 2C; mean ± standard deviation: HC: 10.0 ± 2.5 ms; LC: 13.5 ± 3.1 ms; paired *t*-test, *p* = 5 × 10^−17^, *n* = 45), whereas spike autocorrelations did not show a significant difference in width between LC and HC (Figure 2D; HC: 8.4 ± 2.8 ms; LC: 8.5 ± 3.8 ms; paired *t*-test, *p* = 0.46, *n* = 45), thus confirming that spike timing variability is primarily due to across-trial event-timing variability (see Figure 1). This result was found not only with natural movies, but also with spatiotemporal white noise visual stimulation (Figure S3), and was not related to the cell X or Y type (Figure S4) nor to the occurrence of LGN bursts (Figure S5). These results indicate that decreasing the overall contrast increases the timing variability of groups of spikes (events), but preserves the relative inter-spike timing precision within each group of spikes (as illustrated in Figure 1B, bottom right), at a time scale on the order of ∼10 ms.

The analyses reported above involved the global level of contrast in the full movie (HC versus LC). To further elucidate the relationship between spike timing precision and contrast, we computed the local contrast experienced by each cell as the visual stimulus unfolds in time. Local values of spatiotemporal contrast ranged from 6% to 50% root-mean-squared (RMS) contrast (see Methods). We classified each firing event as corresponding to one in four levels of local contrast: 6–13%, 13–20%, 20–34%, and 34–50%. For each of these contrast levels, we computed the PSTH autocorrelation and spike autocorrelation of each cell, as above. As shown in Figure 2E and 2F, the results presented above for two global levels of contrast (HC and LC) were confirmed with four levels of local contrast. The width of the PSTH autocorrelation significantly decreased as contrast increased (Figure 2E; paired *t*-test; left to right pairwise comparisons: *p* = 1 × 10^−6^, *p* = 1 × 10^−7^, *p* = 2 × 10^−4^, *n* = 45 cells), while spike autocorrelation did not show a significant difference in width across the four different levels of local contrast (Figure 2F; paired *t*-test, *p* > 0.05 in all pairwise comparisons, *n* = 45 cells).

**Figure 3:**
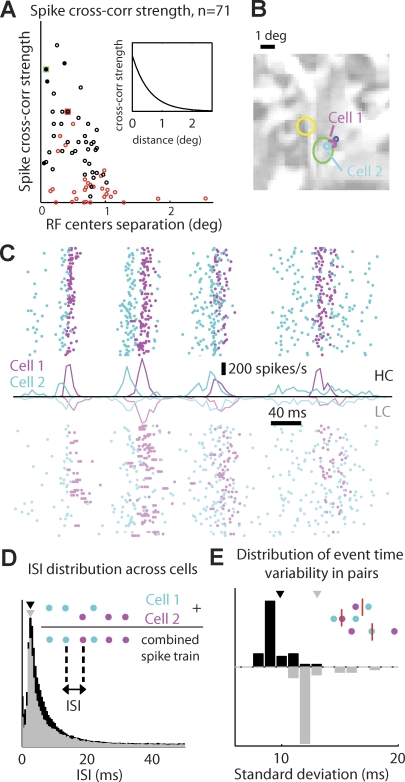
Spike Timing Precision across Cells (A) Correlation strength as a function of the spatial separation between the receptive fields of cell pairs. Each symbol represents a pair of cells (*n* = 71). Black symbols denote pairs included in the pairwise analysis (*n* = 41), whereas red symbols denote pairs excluded from further analysis (see Materials and Methods for inclusion criteria). Inset: Spatial correlation profile of the natural scene stimuli. (B) Receptive fields of five cells recorded simultaneously in a single penetration, superimposed (to scale) on a single frame of the visual stimulus. (C) Response of two of these cells during a 500-ms segment of the movie. (D) Distribution of pairwise ISIs (as defined for the combined spike train in inset) over all included pairs (*n* = 41; see above), at HC (black) and LC (gray). (E) Distribution of pairwise event timing variability (after matching events that overlapped in both cells) for all included cell pairs.

If the relative timing of spikes is preserved at different levels of contrast in single cells, what does it imply across the population? The activity of local groups of cells with neighboring receptive fields can be significantly correlated if the visual input itself has strong spatial and temporal correlations, as is the case with natural scenes [18–20]. Although it had been proposed that retinal and/or LGN neurons could remove these correlations through high-pass filtering achieved by lateral inhibition [21,22], more recent neurophysiological studies suggest that the cells do not de-correlate their inputs [13,23], and thus significant correlations from natural scenes remain present. The strength of pairwise correlation, defined as the area under the cross-correlation function (in the HC condition), decreased with the distance between the receptive fields of two cells (Figure 3A), following the decrease of spatial correlation strength in the visual stimulus (Figure 3A, inset). We focused our analysis on pairs of cells that displayed sufficient cross-correlation in the HC condition (*n* = 41, see Figure 3A) due to strong correlations in their visual input. Neurons with partially overlapped receptive fields (Figure 3B) typically receive similar visual input and therefore tend to share response events, as is evident in the two typical LGN X ON cells shown in Figure 3B and 3C. Both cells tended to fire during the same events, but there was some degree of timing variability (see also Figure S6).

The increased variability in event timing in the LC condition for individual neurons, reported above (see Figure 2), could coexist with a range of effects across a population of neighboring cells, involving within-trial and across-trial variability in the relative timing of spikes from several cells. Since the above result indicates that within cells, event times are more variable across trials at lower stimulus contrast, we then tested the hypothesis that event timing across cells, both within trial and across trials, is also more variable at lower stimulus contrast. From the perspective of a downstream layer 4 V1 neuron receiving direct thalamic input, incoming spike trains that arrive simultaneously from a pair of LGN neurons may be represented by superimposing both spike trains into a single combined spike train. The ISI distribution for these combined spike trains is largely invariant to changes in contrast and peaks at 2.5 ms for both levels of contrast (Figure 3D; full width at half-height: 6 ms at HC, 4 ms at LC), as was the case for single-cell spike trains (Figure 1C). The across-cell event-time variability can be estimated by merging events from both cells that overlap in time and measuring the variability in the median time of the combined event (Figure 3E; mean ± standard deviation of the event-time variability: 9.9 ± 1.0 ms at HC, 13.0 ± 1.4 ms at LC; *n* = 41 cell pairs). Across-cell event-time variability ranged between 8 and 19 ms, larger than but still on the same order of magnitude as that for single cells (Figure 1D). Its average value was slightly higher in the LC than in the HC condition (by 3.1 ms on average across cell pairs; *p* < 0.01, *n* = 41). However, it should be noted that this measure only indicates how the timing of combined firing events varies across repetitions of an identical stimulus. As argued above, it is the precision of spiking across neurons on single trials that is relevant for the neural population code.

**Figure 4:**
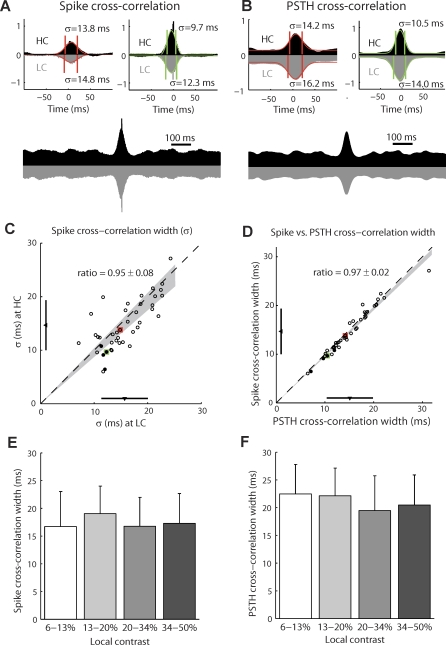
Spike Timing Precision across Cells Is Coarser than within Cells and Is Largely Insensitive to Contrast (A) Spike cross-correlation for pairs of simultaneously recorded cells that exhibited correlated activity. Same conventions as in Figure 2B. Top left: Typical pair of X ON cells. Top right: Pair consisting of a Y OFF cell and an X OFF cell that received common retinal input. Bottom: Average spike cross-correlation over all 41 pairs. (B) PSTH cross-correlation for pairs of simultaneously recorded cells that exhibited correlated activity. Same conventions as in Figure 2A. Top: Same example pairs as in (A). Bottom: Average PSTH cross-correlation over all 41 pairs. (C) Temporal width of the spike cross-correlation for all included cell pairs (*n* = 41). Same conventions as in Figure 2D. (D) Comparison of the PSTH and spike cross-correlations at HC. In (C and D), black dots correspond to pairs sharing common retinal input and colored dots correspond to the two example pairs from (A and B). (E) Temporal width of spike cross-correlation for all included cell pairs, computed on the firing events for which both cells in the pair experienced the same level of local contrast. Each bar represents the mean + 1 standard deviation (*n* = 41 cell pairs) at the corresponding range of local contrast. (F) Temporal width of PSTH cross-correlation for all included cell pairs (*n* = 41), computed on the firing events for which both cells in the pair experienced the same level of local contrast. Each bar represents the mean + 1 standard deviation.

To quantify further the relative precision of spiking across the neuronal population, we computed cross-correlation in pairs of cells. While all pairs under study displayed stimulus-induced correlation, a few pairs also showed correlation on a finer time scale (<1 ms), suggesting that they received common input from the same retinal ganglion cell [15]. Figure 4A and 4B shows the spike and PSTH cross-correlation functions for a pair of cells that shared input from the same retinal afferent (as in 4/41 pairs) and a pair of cells that did not. Importantly, since we are focusing on the neural representation of the visual scene rather than the details of the synaptic connectivity of LGN populations, our measure of spike correlation incorporates “signal” correlations (inherited from correlations present in the visual stimulus) as well as “noise” correlations (arising from other sources such as shared input from a common retinal afferent). Both are integral components of the neural code in natural viewing conditions [24] and, taken together, reflect the relationships between the correlation structure of the visual scene and the functional properties of the local neuronal circuit. Across all pairs of cells under study, the temporal width of spike cross-correlation only showed a small difference between HC and LC (Figure 4C; mean ± standard deviation: 14.7 ± 4.7 ms at HC, 15.7 ± 4.3 ms at LC; *t*-test, *p* = 0.05, *n* = 41 pairs; see also control analyses in Figures S3–S5). Moreover, the PSTH cross-correlation was very similar to spike cross-correlation (Figure 4D). To investigate how spike timing precision of cell pairs is influenced by local visual contrast, we computed the spike and PSTH cross-correlation at different contrast levels: 6–13%, 13–20%, 20–34%, and 34–50%, as done previously for individual cells. The cross-correlation was based only on the events for which the local contrasts in both receptive fields were at the same level. Consistent with the results presented above, there was no trend in the width of spike and PSTH cross-correlations as a function of local contrast (Figure 4E and 4F; some of the pairwise *t*-tests showed statistical significance, but not as a monotonic decrease in correlation width with increasing contrast). These results indicate that within-trial spike timing precision across cells is invariant to the change in contrast of the natural scene, despite the increased variability in event timings for individual cells across trials with decreasing contrast.

**Figure 5:**
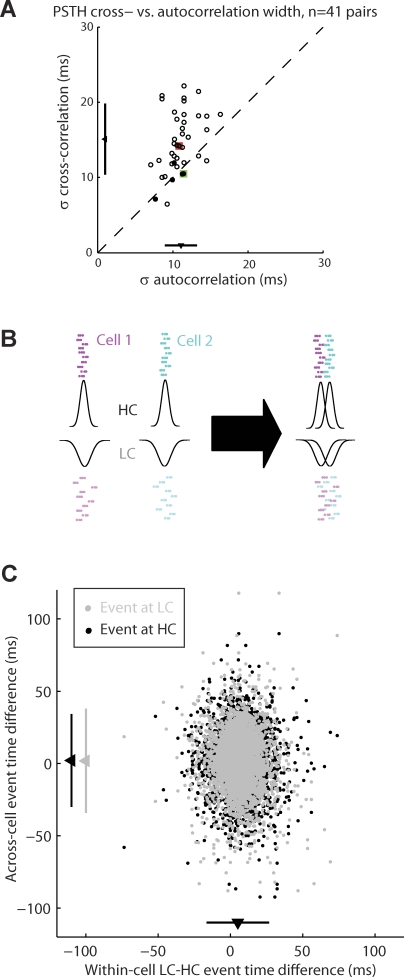
Spike Timing Precision across Cells Is Insensitive to Contrast Despite Contrast Effects on Single-Cell Event Timing (A) Temporal width of the PSTH cross-correlation of cell pairs as a function of the larger temporal width of the PSTH autocorrelations of both cells in each pair at HC. Black dots correspond to pairs sharing common retinal input and colored dots correspond to the two example pairs from Figure 4A and 4B. (B) Spike timing precision across cells is insensitive to the global level of contrast (see main text). (C) Full distribution of event time differences in all pairwise events (*n* = 4,205). The abscissa corresponds to the single-cell, contrast-based event time difference. The ordinate corresponds to the across-cell event time difference, both at HC (black) and at LC (gray). The mean ± 2 standard deviations are represented along each axis by an arrowhead and bar.

As evident in Figures 2F and 4E, the spike cross-correlation obtained from pairs of neurons was consistently wider than the spike autocorrelation obtained from each individual neuron. The difference in width was 8 ms on average (two-sided Wilcoxon rank sum test, *p* < 1 × 10^−6^ for each of four contrast levels, *n* = 45 cells, *n* = 41 cell pairs) and was also found on a pair-by-pair basis. In almost all cell pairs, cross-correlation width was significantly greater than the width of the autocorrelation functions of both cells, as shown in Figure 5A (see Figure S8 for the case of pairs lying close to the unity line, i.e., with similar within-cell and across-cell precision). This finding indicates that spike timing precision was coarser in neuronal pairs than in individual neurons. This decrease in precision can be explained by the fact that, in general, the events are not perfectly aligned across both cells, as illustrated in Figure 5B. Even if cells have wider PSTH events at LC than HC, the overall increase in event time variability from HC to LC by 3 ms is small in the face of pairwise variability, which is on average 8 ms greater than single-cell variability.

Another way to compare pairwise variability with contrast-based variability is by computing, for each shared event, the difference in event time between both cells (and its variability) and the difference in event time between the HC and LC condition (and its variability). As shown in Figure 5C, the difference in event times between two cells is more variable (i.e., has a wider distribution) than the difference in event times between HC and LC. The standard deviations of the distributions across all events are 16 ms (HC) and 18 ms (LC) across cells, and only 11 ms across contrast (*n* = 4,205 events). In other words, the variability in event timing across cells is approximately 1.5 times larger than the variability in event timing across levels of contrast. Therefore, in the face of across-cell variability, the smaller changes in variability due to changes in contrast are negligible. Thus, spike timing precision across most neighboring cells is relatively insensitive to contrast.

## Discussion

In response to movies of natural scenes, spike timing precision across LGN relay cells remained on the order of ∼10 ms, irrespective of contrast. The absolute timing of LGN firing events changed from trial to trial, and more so at low contrast than at high contrast, but the relative timing of spikes occurring in the same trial was insensitive to changes in stimulus contrast—not only within cells but also across correlated neighboring cells. While it is well known that the response properties of single cells are strongly modulated by contrast adaptation, which has effects including slower temporal dynamics and increased gain and selectivity at lower contrast [17,25,26], our results indicate that the temporal precision of the LGN population code is globally maintained in the face of a reduction in contrast.

Interestingly, while in individual neurons PSTH autocorrelations were consistently wider at low contrast than high contrast (Figure 2C and 2E), the width of the PSTH (and spike) cross-correlation was independent of contrast (Figure 4C, 4E, and 4F). This surprising result can be explained by the large variability in event timing across populations of neurons, which is about 1.5 times larger than the variability in event timing caused by changes in contrast (Figure 5C). A related finding is that while in individual neurons, the PSTH autocorrelations were consistently wider than the spike autocorrelations, in cell pairs, PSTH and spike cross-correlation had very similar widths (Figure 4D and 4F). This finding suggests that correlations between cells arose mostly from correlations present in the visual input in our experimental conditions, and that neural “noise” (or across-trial variability arising from intrinsic properties of the system) shows little correlation across neurons (Figure S7). Nevertheless, it should be noted that the presence of weak, pairwise noise correlations does not rule out the possibility of stronger, higher-order correlations at the population level [27–29].

Downstream from the LGN, the influence of stimulus contrast on the timing of spikes across V1 cells has only been recently addressed. Spike timing precision across cells in anesthetized macaque V1 reportedly decreased for low-contrast grating stimuli [10], whereas we found that it was contrast-independent in the cat LGN for natural stimuli. Further, a recent study in cortical slices suggested that the degree of noise correlation between two neighboring cortical cells increased with firing rate [30], unlike the thalamic signal and noise correlations reported here that were invariant to contrast-driven changes in firing rates. These discrepancies may be attributable to differences in the experimental preparations and in the visual stimuli, or they could be explained by specific contrast adaptation mechanisms that occur in cortex but not in precortical areas. For example, in the peripheral auditory system, adaptation to a constant stimulus reduces the firing rate but does not impair spike timing precision [31], in a similar fashion as what we found in the visual thalamus.

Preserving spike timing across cells at a ∼10-ms resolution may be a crucial aspect of the neural population code in natural conditions, given that the representation of spatiotemporally varying natural scenes requires a finer temporal precision than the time scale of the visual stimulus [12]. Furthermore, a 10-ms temporal resolution could facilitate “temporal coding” under the hypothesis that the neural representation of sensory information relies on specific temporal patterns of spikes [32–34]. However, the existence or preservation of specific temporal patterns is beyond the scope of the present study. Downstream from the thalamus, spike timing precision may well vary along the visual pathway. Single-cell studies found that trial-to-trial variability is similar in the LGN and V1 when a V1 cell is presented with its preferred stimulus, but that V1 cells become more variable for suboptimal stimuli [5,8,35]. In the presence of natural movies, which combine optimal and nonoptimal stimulation for each cell, recent studies in primate V1 indicate that some visual information is present in the phase of local field potentials at low frequency (<12 Hz) [36] and that power associated with spiking activity is only informative at frequencies under 12 Hz [37]. Therefore, the relevant time scale in V1 is probably on the order of tens of milliseconds, only slighter longer than what we found in the LGN. The small increase in variability in V1 trial-to-trial spike timing compared to the LGN [35] may be explained by nonlinearities in the spiking mechanism and may coexist with lower variability in V1 membrane potential [38]. It is also possible that temporal precision is higher between cortical cells receiving input from geniculate cells that share a common retinal afferent, in a divergent–convergent pattern of connectivity. In any case, it is difficult to relate these previous results to population coding. The degree of spike timing precision across V1 cells, especially in natural viewing conditions, is not well quantified, and how it would be affected by contrast or other variables is unknown. Further studies are needed to elucidate how the functional architecture of the thalamocortical circuit constrains spike timing precision across cells and how it affects the neural code entering V1.

Preserving synchrony across cells could have a number of functional advantages. Synchronous spikes from several thalamic neurons are reportedly needed to drive cortical cells to threshold [15,39]. Recent studies have suggested that the cortical response is sensitive to the timing of thalamic inputs and that the “window of opportunity” for integration of excitatory inputs at the thalamocortical synapse remains unchanged in the face of adaptation [40]. Therefore, the relative timing of spikes in thalamic neurons could be an important aspect of the population neural code entering primary sensory cortices and could benefit from being insensitive to some properties of the sensory world while maintaining sensitivity to other, presumably more interesting, features.

## Materials and Methods

### Neural recordings.

Single-cell activity was recorded extracellularly in the LGN of anesthetized and paralyzed cats using a seven-electrode system. Four animals were used for a total of ten electrode penetrations. Surgical and experimental procedures were performed in accordance with United States Department of Agriculture guidelines and were approved by the Institutional Animal Care and Use Committee at the State University of New York, State College of Optometry. As described in [41], cats were initially anaesthetized with ketamine (10 mg kg^−1^ intramuscular) followed by thiopental sodium (20 mg kg^−1^ intravenous during surgery and at a continuous rate of 1–2 mg kg^−1^ h^−1^ intravenous during recording; supplemented as needed). A craniotomy and duratomy were performed to introduce recording electrodes into the LGN (anterior, 5.5; lateral, 10.5). Animals were paralyzed with atracurium besylate (0.6–1 mg kg^−1^ h^−1^ intravenous) to minimize eye movements, and were artificially ventilated. Geniculate cells were recorded extracellularly from layer A of LGN with a multielectrode matrix of seven electrodes [42]. The multielectrode array was introduced in the brain with an angle that was precisely adjusted (25–30 degrees antero-posterior, 2–5 degrees lateral-central) to record from iso-retinotopic lines across the depth of the LGN. A glass guide tube with an inner diameter of ∼300 μm at the tip was attached to the shaft probe of the multi-electrode to reduce the inter-electrode distances to approximately 80–300 μm. Layer A of LGN was physiologically identified by performing several electrode penetrations to map the retinotopic organization of the LGN and center the multielectrode array at the retinotopic location selected for this study (5–10 degrees eccentricity). Recorded voltage signals were conventionally amplified, filtered, and passed to a computer running the RASPUTIN software package (Plexon). For each cell, spike waveforms were identified initially during the experiment and were verified carefully off-line by spike-sorting analysis. Cells were classified as X or Y according to their responses to counterphase sinusoidal gratings. Cells were eliminated from this study if they did not have at least 2 Hz mean firing rates in response to all stimulus conditions, or if the maximum amplitude of their spike-triggered average in response to spatiotemporal white noise stimuli was not at least five times greater than the amplitude outside of the receptive field area.

### Visual stimulation.

For each cell in the main experiments, visual stimulation consisted of 128–240 repeats of one of two short movies of natural scenes taken from “cat-cam” movies recorded from a small camera mounted on top of a cat's head while roaming in grasslands and forests [16]. As in [17], to improve temporal resolution, movies were interpolated by a factor of two (from 25 to 50 Hz) using commercial software (MotionPerfect, Dynapel Systems Inc.) and then presented at 60 frames per second, i.e., at 1.2× speed. Following interpolation, the intensities of each movie frame were rescaled to have a mean value of 125 (where the full range of intensity values was 0–255). Each movie spanned 48 × 48 pixels at an angular resolution of 0.2 degree per pixel. The first movie (presented to 28 of the cells included in the final analysis) was 750 frames and lasted 12.5 s, while the second movie (presented to the remaining 17 cells) was 600 frames long and lasted 10 s. The stimuli were presented at 60 frames per second with a 120-Hz monitor refresh rate, such that each frame was displayed twice. Each movie was repeated 64–120 times at each of two global levels of luminance contrast: 0.4 (high contrast, or HC) and 0.15 (low contrast, or LC) RMS contrast [17].

In addition to “cat-cam” natural movies, as a control for each cell we also used visual stimulation consisting of spatiotemporal binary white noise shown at high contrast (0.55 RMS contrast) and low contrast (0.20 RMS contrast). The spatial resolution and refresh rate of the white noise stimulus were the same as those of the natural scene movies. Each cell in the reported data was stimulated with the natural scenes movies as well as the white noise stimuli with an equal number of repeats (120 repeats at each level of contrast for 28/45 cells, 64 repeats at each level of contrast for 17/45 cells).

### Receptive fields.

For each cell, the spatiotemporal receptive field was estimated by standard spike-triggered-average techniques based on spatiotemporal white noise stimuli [43,44]. The spatial receptive field was fitted with a difference of two-dimensional Gaussians. The distance between receptive fields was defined as the distance between the centers of the Gaussians. The diameter of each receptive field was estimated as the average length of the major and minor axes of the one–standard deviation ellipse that defines the receptive field center. The overlap between two receptive fields was evaluated as the normalized dot product of the two receptive fields, computed after each receptive field had been normalized so that its dot product with itself was one [45,46].

### Properties of neural response.

For each cell at each level of contrast (HC or LC), a single PSTH was computed as the cumulative response of the cell over all 64–120 repeats of the same short movie. Each PSTH was therefore 10 or 12.5 s long, depending on the duration of the stimulus presented to the cell. ISIs were computed as the time intervals between consecutive spikes; in the case of pairs of cells, we merged the spike trains from both cells and computed the ISIs from the combined spike train. Bursts were identified as groups of spikes separated from each other by 4 ms or less, where the first spike is preceded by a period of silence of 100 ms or more [47–49]. The degree of burstiness exhibited by each neuron was defined as the percentage of spikes belonging to a burst.

### Correlation analysis.

Previous studies typically define temporal precision of single neurons as the standard deviation of the spike times within an identified event across trials [6–9,31,35]. In this study, we first defined a related measure which is the (temporal) width of the central peak in the PSTH autocorrelation [50]. The width of PSTH events and the width of the PSTH autocorrelation function are directly related, by a factor of √2 in the Gaussian approximation. In computing the PSTH (and its autocorrelation), all spike trains that the cell produced in response to multiple repeats of an identical stimulus were collapsed into one “lumped” spike train (i.e., a PSTH with a 1-ms bin size, of the same duration as a single presentation of the movie, i.e., 10 or 12.5 s). In the PSTH autocorrelation measure, the relative timing of spikes within a given trial or across all trials were confounded. To investigate within-trial temporal precision, we therefore computed a different measure: the width of the central broad peak in the spike autocorrelation, which we defined as the autocorrelation function of the full (several minutes long) spike train without collapsing the trials together [51,52].

Although analysis of single cells was a necessary first step, the primary focus of this study was on spike timing variability across cells. Two definitions of cross-correlation were used: spike cross-correlation [52,53] and PSTH cross-correlation, which is the cross-correlation between two PSTHs. Spike cross-correlation width gives the spike timing variability across cells within each trial. PSTH cross-correlation has a different meaning: it is approximately equivalent to the “shuffled” or “shifted” spike correlation, in which each spike train of one cell is paired with a spike train of the other cell recorded during a different repeat of the same stimulus. The PSTH cross-correlation averages correlations from all possible pairwise combinations of repeats (actually including the non-shuffled one, which is only one in thousands of combinations and therefore has a negligible contribution).

All four types of correlation functions (spike or PSTH, auto- or cross-correlation) were made analogous to Pearson's correlation coefficient by (i) subtracting the product of the average firing rates, and (ii) dividing by a normalization factor (see below), such that correlation could take values between −1 and +1. To determine the existence of a central peak or trough in a correlation function, we found the Gaussian function that best fit the central ±100 ms, in a least-mean-square sense. The standard deviation of this Gaussian provides a measure of the correlation width. In the case of autocorrelation, the height *A_i_* of the best-fitting Gaussian was measured for each cell *i* and was subsequently set to 1 to normalize the autocorrelation function. In the case of cross-correlation between cells *i* and *j*, the best-fitting Gaussian was normalized by a factor of 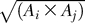 , where *A_i_* and *A_j_* are the heights of each respective autocorrelation function before normalization. The area under the Gaussian curve after normalization was used to define the strength of the cross-correlation between two neurons.

*Inclusion criterion for pairs*: A pair of cells was included in the final pairwise analysis if its spike cross-correlation function peaked at a value of 0.065 or higher, an arbitrary threshold below which the cross-correlation function could not be well fitted by a Gaussian function.

### Correlations in the visual stimulus.

For all pairs of pixels corresponding to the receptive field centers of pairs of cells, we measured the correlation function between both time series (i.e., the time series of the intensity values of each pixel across all frames of the movie). Correlation strength was defined as the area under the Gaussian curve that best fit the cross-correlation function. The resulting spatial profile of correlation in the visual input, i.e., the graph of correlation strength as a function of the distance between two pixels, was fitted (in the least-mean-square sense) to an exponential function with a negative exponent, which is the form expected for spatial correlations in a signal with a power spectrum decreasing as 1/*f* ^2^ with spatial frequency.

### Event analysis.

*Single-cell event analysis*: PSTH “events” were first defined in the PSTH at HC as times of firing interspersed with periods of silence lasting at least 20 ms. If the standard deviation of all spike times constituting an event was less than 20 ms, an attempt was made to break up the event into several events, a procedure in which the spikes were fitted to a mixture-of-Gaussians model using the Expectancy Maximization (EM) algorithm for maximum likelihood [54]. PSTH events at LC were then defined by aligning LC spikes to existing HC events if possible, with a preference for an HC event that occurred earlier rather than after the LC spike (since it is known that spikes tend to be more delayed at LC than HC). If no corresponding HC event was found, a new event was created at LC, with a corresponding empty event at HC. The timing of an event on a given repeat was defined as the median time of all spikes composing this event. For each event at a given contrast level, the event time variability was the standard deviation of the timing of the event across repeats. We computed for each cell its average event time variability across all events.

*Pairwise event analysis:* Starting from the single-cell event analysis above, each event from the first cell was matched to one or several events in the second cell with which it overlapped in time. If several events in one cell could be matched to a single event in the other cell, these events were merged into one. The list of all events that could be matched across the two cells constituted the list of “shared events.” For each shared event at a given contrast level, the event time variability was the standard deviation of the timing of the event across repeats and across both cells. We computed for each cell pair its average event time variability across all events.

*Event-by-event analysis of event time difference, within cells and across cells:* For all pairs (cell A and cell B), for each pairwise event that existed in the four cases (cell A at HC, cell A at LC, cell B at HC, and cell B at LC), we computed within-cell HC-LC event time difference as the average event time at LC minus the average event time at HC, for each of the two cells (cell A and cell B). In other words, we hold the cell fixed and varied across two contrast levels. We also computed across-cell event time difference at a given contrast level (HC or LC) as the average event time for cell A minus the average event time for cell B. In this case, we hold contrast fixed and varied across two cells. Therefore, each pairwise event yielded four different data points (2 × 2) to compare the distributions of across-cell and within-cell event time difference, as shown in Figure 5C.

### Local spatiotemporal contrast.

For each cell, we computed the local value of spatiotemporal contrast as follows. For each firing event determined as above, we identified the smallest rectangle in the image that encompassed the cell's receptive field (e.g., 3 × 4 pixels) and extracted from the movie the luminance values of these pixels at the six previous frames. Six movie frames at 60 Hz correspond to a duration of ∼100 ms, matching the temporal kernel of the cells. The RMS contrast of this spatiotemporal patch of the movie was computed as the standard deviation over all the corresponding pixel values (e.g., 3 × 4 × 6 values). In the LC movie, local contrast values in the 45 cells ranged from 6–20% RMS contrast. In the HC movie, they ranged from 14–50%. For each cell, each firing event (in either the HC or LC condition) was assigned one in four levels of local contrast: 6–13%, 13–20%, 20–34%, or 34–50%. Correlation analysis was then performed as described above on small sections of data corresponding to individual events. We restricted the cross-correlation analysis to the firing events for which both cells experienced a value of local contrast that fell into the same range (out of the four ranges defined above).

## Supporting Information

Figure S1 Distribution of Firing Rates and Latencies (A) Distribution of average firing rates at HC versus LC (*n* = 45 cells). The dashed line is the unity line. The shaded area indicates the 95% confidence estimate of the mean HC/LC ratio of the average firing rates. The mean ± 1 standard deviation are represented along each axis by an arrowhead and bar.
(B) Distribution of latencies between LC and HC events, across all 7,878 events from 45 cells. Latency is defined to be positive if LC occurs later than HC. The distribution of latencies has long tails and is only shown for the central ±50 ms. The mean and standard deviation of the best-fitting Gaussian (in red) are indicated.
(258 KB PDF)

Figure S2 Connections between Spike Train Properties and Correlation Measures (456 KB PDF)

Figure S3 Results of Experiments in Which Visual Stimulation Consisted of Spatiotemporal White Noise Stimuli (A) Temporal width of the PSTH autocorrelation at HC versus LC for the same 45 cells as in the main results. This figure has the same conventions as in Figure 2C.
(B) Temporal width of the spike autocorrelation at HC versus LC for the same 45 cells. Same conventions as in Figure 2D.
(C) Temporal width of the spike cross-correlation at HC versus LC for the subset of pairs that exhibited a measurable amount of cross-correlation with white noise stimuli. Same conventions as in Figure 4C.
(271 KB PDF)

Figure S4 Control for X versus Y Cell Type The main single-cell results (see Figure 2) hold for both X cells (red) and Y cells (green). Cells of unknown X or Y type are represented in black. The shaded areas correspond to the 95% confidence estimates of the mean HC/LC ratio of the autocorrelation widths, for X cells (red, *n* = 19) and Y cells (green, *n* = 18). This figure has the same conventions as in Figure 2C and 2D.
(A) Temporal width of PSTH autocorrelation for natural movies.
(B) Temporal width of spike autocorrelation for natural movies.
(C) Temporal width of PSTH autocorrelation for spatiotemporal white noise stimuli.
(D) Temporal width of spike autocorrelation for spatiotemporal white noise stimuli.
(344 KB PDF)

Figure S5 Control for the Occurrence of LGN Putative Calcium Bursts (A) Temporal width of the PSTH autocorrelation at HC versus LC (as in Figure 2C), for the 34 least bursty cells (out of 45 cells total).
(B) Temporal width of the spike autocorrelation at HC versus LC (as in Figure 2D), for the same 34 least bursty cells.
(C) Temporal width of the spike cross-correlation at HC versus LC, as in Figure 4C, for the 22 pairs (out of 41 pairs in the main analysis) involving the 34 least bursty cells from (A) and (B).
(281 KB PDF)

Figure S6 Examples of Raster Plots and PSTH for a Single Firing Event Shared by Two Cells (A) Event shared by two X ON cells recorded simultaneously, showing raster plots and PSTHs. Only the repeats in which cell 1 fired at least one spike are shown (56/64 repeats at LC, 63/64 at HC).
(B) Same event for cell 1 only. The trials have been reordered based on the event time (calculated as the median spike time within the event for each trial, denoted with black circle; see Materials and Methods). The black lines are the best linear fit to the event times, excluding the ten repeats on which the cell fired the earliest and the ten repeats on which the cell fired the latest.
(C) Same data as in (A) where the trials are sorted as in (B) by increasing event time for cell 1.
(D) Same data as in (A) where the trials are “dejittered” by aligning their median event time for cell 1.
(E) Another event shared by two other cells from a different electrode penetration (cell 3 spikes: red circles; cell 4 spikes: small blue dots). These two cells, an X ON cell and a Y ON cell, share input from the same retinal afferent.
(F) Same as (E) except that the trials are sorted by increasing event time for cell 3. Only the repeats in which cell 3 fired at least one spike are shown (60/120 repeats at LC, 44/120 at HC).
(485 KB PDF)

Figure S7 Distribution of Noise Correlation The distribution is across all pairs included in the analysis. The mean and standard deviation are indicated.
(176 KB PDF)

Figure S8 Control for Pairs of Cells with the Most Receptive Field Overlap, Which Are Likely to Fire at Similar Event Times (A) Difference in width between the spike cross-correlation function and the wider of the two spike autocorrelation functions for each pair, as a function of the distance between the two receptive fields. Circles: 15 most closely-spaced pairs. The circles filled in black represent the four pairs of cells receiving common retinal input, as in Figure 4C. Crosses: 26 remaining pairs.
(B) Temporal width of the spike cross-correlation at HC versus LC for the 15 pairs shown by circles in (A) (*n* = 15 pairs). Same conventions as in Figure 4C.
(284 KB PDF)

Text S1 Supporting Analyses (122 KB PDF)

## Abbreviations

HC: high contrast
ISI: inter-spike interval
LC: low contrast
LGN: lateral geniculate nucleus
PSTH: peri-stimulus time histogram
RMS: root-mean-squared
