# Supplementary material for: Timing Precision in Population Coding of Natural Scenes in the Early Visual System
Source: PLoS Biol. 2008 Dec 16;6(12):e324. doi: 10.1371/journal.pbio.0060324 (PMC2602720; doi:10.1371/journal.pbio.0060324)
Supplement: Supplementary file 1 [file pbio.0060324.sd001.pdf]

Text S1. Supporting Analyses

Desbordes et al., “Timing Precision in Population Coding of Natural Scenes in the Early Visual System”

This Supporting Text provides extended analyses reinforcing the main points of the work. We show the distributions of firing rates and latencies in all cells (Fig. S1). We illustrate our correlation measures (Fig. S2) and then provide control analyses that support our results for the case of spatiotemporal white noise stimuli (Fig. S3), X vs. Y-type cells (Fig. S4) and cells exhibiting a low number of putative calcium bursts (Fig. S5). We also provide several detailed examples of a PSTH event shared by two cells (Fig. S6). We then discuss noise vs. signal correlations (Fig. S7). Finally, we show control analyses that support our results for the case of pairs of cells that share very similar event times (Fig. S8).

Figure S1: Distribution of Firing Rates and Latencies

Figure S1A shows each cell’s firing rate at HC vs. LC. The ratio of HC firing rate to LC firing rate across all cells is significantly greater than 1 (mean: 1.30), meaning that the firing rate was 30% higher at HC than LC (or 23% lower at LC than HC). Figure S1B shows the distribution of latencies across cells, computed as follows. First, firing events were identified in each cell at HC and corresponding events at LC were determined (see Methods). Second, whenever a firing event existed at both HC and LC, the mean time of the first spike in the event was computed at HC and LC, and the difference was the latency for this event (such that the latency is positive if LC occurs later than HC). The mean (3.4 ms) was significantly different from 0 (t-test, $p<0.001$).

Figure S2: Connections between Spike Train Properties and Correlation Measures

Figure S2 provides a schematic illustration of the four measures of correlation used in this study. Figure S2A illustrates the two different measures of autocorrelation (PSTH autocorrelation and spike autocorrelation) in the hypothetical case of a single cell producing a single PSTH event. The correlation functions are only shown for the central ± 100 ms. The PSTH autocorrelation, as shown in the left panel, confounds within-trial and across-trial spike timing variability. To distinguish them, one can think of the variability in the timing of the spikes as having two origins: across-trial variability in event timing (Fig. S2A, right, top) and within-trial variability in spike timing (Fig. S2A, right, bottom). The latter corresponds to the width of the spike autocorrelation defined above.

Figure S2B illustrates the PSTH and spike cross-correlation for two hypothetical extreme cases of pairs of cells. In the example on the left, the two cells are conditionally independent, and thus the event timing on each trial is uncorrelated. In this case, shuffling

repeats has no effect and the PSTH cross-correlation and spike cross-correlation are the same. In the example on the right, the two cells are strongly correlated on a trial-by-trial basis, and shuffling trials destroys this correlation. Therefore, the spike cross-correlation (which reflects the within-trial correlations) is significantly narrower than the PSTH cross-correlation. In fact, if the cells are strongly correlated in this sense (which can happen for two LGN cells receiving common input from the same retinal ganglion cell), their spike cross-correlation will be very similar to the wider of the two spike autocorrelations.

Figure S3: Control Analyses with White Noise Stimuli

We repeated all experiments using *spatiotemporal white noise* as visual stimulation, using the same spatial and temporal scale as in the main experiments (0.2 degree per pixel, 60 Hz frame rate, 120 Hz monitor rate). The results with white noise were analogous to those with natural scene stimuli. PSTH autocorrelation width was significantly greater at LC than HC (Fig. S3A; p=5.3e-17, n=45). Spike autocorrelation width was slightly greater at HC than LC (Fig. S3B; p=0.02, n=45), which is likely due to the fact that the low-contrast white noise stimulus did not induce many spikes in a given single trial, and therefore the spike correlation functions were more noisy and less well fit by a Gaussian in this stimulus condition. It should be noted that the number of pairs of cells displaying sufficient pairwise correlation was lower in this case (15 pairs, instead of 41 pairs with natural scenes) due to the lack of correlation in the visual stimulus. Only pairs with significant receptive field overlap receive similar visual input in the case of white noise stimulus. For these 15 pairs, spike cross-correlation width did not vary with contrast (Fig. S3C; p=0.49, n=15).

Figure S4: Control Analyses for Different Cell Types

In Figure S4 we show that our single-cell results do not depend on the *X or Y functional classification* of the cells (where cells were classified as X or Y according to their responses to counterphase sinusoidal gratings). In the case of natural scene stimuli, the PSTH autocorrelation width was significantly greater at LC than HC (Fig. S4A), both for X cells (p=8.2e-9, n=19) and Y cells (p=2.7e-6, n=18), while the spike autocorrelation width was not different at HC and LC (Fig. S4B), neither for X cells (p=0.56, n=19) nor for Y cells (p=0.53, n=18). In the case of spatiotemporal white noise stimuli, similarly, the PSTH autocorrelation width was significantly greater at LC than HC (Fig. S4C), both for X cells (p=2.6e-9, n=19) and Y cells (p=6.1e-11, n=18), while the spike autocorrelation width was not significantly different at HC and LC (Fig. S4D), neither for X cells (p=0.055, n=19) nor for Y cells (p=0.16, n=18).

Figure S5: Control Analyses for LGN Cells Generating Putative Calcium Bursts

Low-threshold voltage-dependent T-type Ca^{2+} channels *bursts* are often observed in the thalamus (Guido & Weyand, 1995; Lu, Guido, & Sherman, 1992; Ramcharan, Cox, Zhan, Sherman, & Gnadt, 2000). We identify bursts by patterns of short inter-spike intervals preceded by long periods of inactivity (see Methods), which we label as putative calcium channel bursts. In Figure S5 we show that the main single-cell and pairwise results (see Fig. 2, C and D, and Fig. 4C) hold irrespective of the amount of bursting exhibited by the cells. Here we only kept the 22 pairs involving the 34 least bursty cells. In these cells, less than 22% of the spikes belonged to a burst, where a burst is defined as a group of two or more spikes, each of which is <4 ms apart, with the first spike preceded by >100 ms of silence (Lesica & Stanley, 2004; Lu et al., 1992). In these cells, PSTH autocorrelation width was significantly greater at LC than HC (Fig. S5A; p=2.6e-12, n=34) while spike autocorrelation width was not different at HC and LC (Fig. S5B; p=0.19, n=34) and spike cross-correlation was independent of contrast as well (Fig. S5C; p=0.56, n=22).

Figure S6: PSTH Events Shared by Two Cells

Figure S6 illustrates two examples of a single PSTH event shared by two cells. Fig. S6A shows the simultaneous activity of two X ON cells (cell 1 receptive field: 0.43 degree diameter; cell 2 receptive field: 0.40 degree diameter; distance between receptive field centers: 0.38 degree; receptive field overlap measured as the normalized dot product: 61%). Fig. S6B focuses on cell 1. The spike rasters are the same as in Fig. S6A, but the trials have been reordered based on the event time (calculated as the median spike time within the event for each trial, shown as a black open symbol). Regression lines were fit to the median spike times in this ordered representation. The scatter of spikes around the regression lines (illustrated figuratively by the small Gaussians) represents the within-trial spike timing variability, which was the same at HC (top) and LC (bottom). However, the variability in the time of occurrence of the whole group of spikes, or across-trial spike timing variability (schematized as the slope of the regression lines) was greater at LC than HC.

In Fig. S6C, trials are sorted as in Fig. S6B with increasing time of events emitted by cell 1. Sorting trials according to the first cell does not reveal any ordering in the spikes of cell 2, neither at HC nor at LC. Similarly, "dejittering" trials by aligning their average event time for cell 1 (Fig. S6D), which has the effect of removing the across-trial variability for this cell, clearly reduces the width at half-height of the PSTH event for this cell (compared with Fig. S6A), but does not affect cell 2. Therefore, while the ability of an ideal observer to "de-jitter" spike trains might improve stimulus decoding – and thus the fidelity of the neural representation – in single cells under certain conditions (see (Aldworth, Miller, Gedeon, Cummins, & Dimitrov, 2005) for a demonstration of this phenomenon in the auditory pathway), our results seem to indicate that this is not the case across a population of LGN cells.

These examples suggest that in general, spikes from different cells can occur at

various times within a shared event, but are constrained within the time window of the event (median event duration across all cells: 32 ms at HC, 31 ms at LC). A notable exception occurs for tightly-correlated cells that shared input from the same retinal afferent (Alonso, Usrey, & Reid, 1996; Usrey, Reppas, & Reid, 1998). In this particular case (shown in Fig. S6, E and F), sorting trials according to one cell (Fig. S6F) makes it more evident that both cells tend to fire spikes precisely at the same time (<1 ms apart).

Figure S7: Signal vs. Noise Correlation

For the majority of pairs in our dataset, the spike cross-correlation and PSTH cross-correlation were very similar (see Fig. 4D). The PSTH cross-correlation is equivalent to a “shuffled” version of the spike cross-correlation, as explained in the Methods. Fig. 4D indicates that direct random shuffling of trials between the two cells yields the same cross-correlation profile as in the un-shuffled case. Within-trial cross-correlations are therefore indistinguishable from across-trial cross-correlations. This is an indication that the measured correlations are mostly due to the “signal”, i.e., the visual stimulus, and that neural “noise” (or across-trial variability arising from intrinsic properties of the system) shows little correlation across neurons. To confirm this finding, we computed the value of noise correlation for each pair, using standard methods (Bair, Zohary, & Newsome, 2001; de la Rocha, Doiron, Shea-Brown, Josic, & Reyes, 2007; Kohn & Smith, 2005). In brief, the PSTH cross-correlation (a.k.a. “all-shuffle predictor”) was subtracted from the spike cross-correlation, and the resulting function was normalized by the square-root of the product of the shuffle-corrected autocorrelation functions of both cells (each being computed, similarly, as the difference between the spike autocorrelation and the PSTH autocorrelation). Noise correlation was defined as the area under the central +/- 400 ms under this curve (referred to as $r_{ccg}(400)$ in (Kohn & Smith, 2005)). The distribution of values for noise correlation is shown in Fig. S7 (0.10 ± 0.11 mean $\pm$ standard deviation).

Note that the fact that noise correlations have low values does not mean that they are negligible from the system’s perspective, since weak pairwise noise correlations do not rule out stronger correlations at the scale of a larger population (Schneidman, Berry 2nd, Segev, & Bialek, 2006; Shamir & Sompolinsky, 2004). It is also possible that weak spike correlations coexist with stronger membrane potential correlations on a broader time scale (Lampl, Reichova, & Ferster, 1999), which may be relevant in multi-cell correlations (or synchrony). A few temporally correlated (or synchronous) spikes from several thalamic afferents converging onto the same cortical cell could be enough to drive the cortical cell even if their synapses have low efficacy, as found in the somatosensory pathway (Bruno & Sakmann, 2006).

Although in our recordings spike cross-correlation and PSTH (or shuffled) cross-correlation were very similar in general, there were a small number of exceptions. The only notable exception was for pairs of tightly-correlated cells, which receive common input from the same retinal ganglion cell (Alonso et al., 1996); there were four such pairs in our analysis. In these pairs the broad correlations were visible in both the spike cross-correlation and the PSTH cross-correlation, but the tight correlation due to common retinal input (on a time scale of 1 ms) was only exhibited in the spike cross-correlation, as can be seen in the

example shown in Fig. 4A.

Interestingly, while our results show that cross-correlation functions – whether shuffled or not – have the same temporal width at HC and LC, in a macaque V1 study shuffled cross-correlograms were wider at LC than HC (Kohn & Smith, 2005). While it is possible that the discrepancy originates from investigating different visual brain structures and different species, it should also be noted that Kohn and Smith used sinusoidal gratings as visual stimuli, and therefore it is unclear how these results apply to spike timing precision under naturalistic visual stimulation, which is what we investigated in this study. In natural conditions, across-cell spike timing precision in V1 could either be contrast-independent, as we found in the LGN, or decrease at low contrast in a similar way that across-trial precision is impaired for suboptimal visual stimuli. Further studies in V1 with natural stimuli are needed to resolve this issue.

Figure S8: Control for Pairs of Cells with Very Similar Event Times

The main findings of this paper are that spike timing variability (i) is generally greater across population of cells than within individual cells and (ii) does not depend on the global level of contrast. These findings are summarized in the illustration of Figure 5B. In this Supporting Figure, we performed a control analysis to explore the case of pairs of cells that tend to fire events at very similar times (*unlike* the schematic example of Figure 5B) and asked whether spike timing variability across cells is also contrast-independent in this case. For these pairs, spike timing variability was not much greater across cells than within each individual cell. In other words, the difference between spike cross-correlation width and spike autocorrelation widths was small. In Fig. S8A we show that this difference increased with the distance between the cells receptive fields (linear regression: $r^2 = 0.24$), i.e., cross-correlation became progressively wider than autocorrelation. This confirms the expectation that cells with closely-spaced, more overlapped receptive fields probably share more events. The fifteen most closely-spaced pairs (circles) showed little difference between their autocorrelation and cross-correlation width (5 ms at most), unlike the more distant pairs (crosses). Tightly-correlated pairs of cells that shared input from the same retinal afferent (filled circles) seemed to show the least difference between autocorrelation and cross-correlation width (less than 2 ms).

To test whether the across-cell variability is contrast-invariant even for pairs of cells with similar event times, we focused our analysis on the fifteen pairs of cells whose receptive fields were separated by the smallest distance (represented with circles in Fig. S8A). This control analysis is reported in Fig. S8B, showing the same result as in Fig. 4C, which is that cross-correlation width was not significantly different at HC and LC (p=0.18, n=15). Since these fifteen pairs are more likely to receive similar visual stimulation, and have a spike cross-correlation not much wider than the wider autocorrelation of the two cells (as shown in Fig. S8A), this control shows that it is not the case that, given that both cells fire at different event times, cross-correlation is simply broader than the autocorrelations of both individual cells at any contrast level.

Supporting References

Aldworth, Z. N., Miller, J. P., Gedeon, T., Cummins, G. I., & Dimitrov, A. G. (2005). Dejittered spike-conditioned stimulus waveforms yield improved estimates of neuronal feature selectivity and spike-timing precision of sensory interneurons. *Journal of Neuroscience, 25*(22), 5323-5332.

Alonso, J. M., Usrey, W. M., & Reid, R. C. (1996). Precisely correlated firing in cells of the lateral geniculate nucleus. *Nature, 383*(6603), 815-819.

Bair, W., Zohary, E., & Newsome, W. T. (2001). Correlated firing in macaque visual area MT: time scales and relationship to behavior. *J Neurosci, 21*(5), 1676-1697.

Bruno, R. M., & Sakmann, B. (2006). Cortex is driven by weak but synchronously active thalamocortical synapses. *Science, 312*(5780), 1622-1627.

de la Rocha, J., Doiron, B., Shea-Brown, E., Josic, K., & Reyes, A. (2007). Correlation between neural spike trains increases with firing rate. *Nature, 448*(7155), 802-806.

Guido, W., & Weyand, T. (1995). Burst responses in thalamic relay cells of the awake behaving cat. *Journal of Neurophysiology, 74*(4), 1782-1786.

Kohn, A., & Smith, M. A. (2005). Stimulus dependence of neuronal correlation in primary visual cortex of the macaque. *Journal of Neuroscience, 25*(14), 3661-3673.

Lampl, I., Reichova, I., & Ferster, D. (1999). Synchronous membrane potential fluctuations in neurons of the cat visual cortex. *Neuron, 22*(2), 361-374.

Lesica, N. A., & Stanley, G. B. (2004). Encoding of natural scene movies by tonic and burst spikes in the lateral geniculate nucleus. *Journal of Neuroscience, 24*(47), 10731-10740.

Lu, S. M., Guido, W., & Sherman, S. M. (1992). Effects of membrane voltage on receptive field properties of lateral geniculate neurons in the cat: contributions of the low-threshold Ca2+ conductance. *Journal of Neurophysiology, 68*(6), 2185-2198.

Ramcharan, E. J., Cox, C. L., Zhan, X. J., Sherman, S. M., & Gnadt, J. W. (2000). Cellular mechanisms underlying activity patterns in the monkey thalamus during visual behavior. *Journal of Neurophysiology, 84*(4), 1982-1987.

Schneidman, E., Berry 2nd, M. J., Segev, R., & Bialek, W. (2006). Weak pairwise correlations imply strongly correlated network states in a neural population. *Nature, 440*(7087), 1007-1012.

Shamir, M., & Sompolinsky, H. (2004). Nonlinear population codes. *Neural Computation, 16*(6), 1105-1136.

Usrey, W. M., Reppas, J. B., & Reid, R. C. (1998). Paired-spike interactions and synaptic efficacy of retinal inputs to the thalamus. *Nature, 395*(6700), 384-387.
