# Supplementary material for: Timing Precision in Population Coding of Natural Scenes in the Early Visual System
Source: PLoS Biol. 2008 Dec 16;6(12):e324. doi: 10.1371/journal.pbio.0060324 (PMC2602720; doi:10.1371/journal.pbio.0060324)
Supplement: Supplementary file 2 [file pbio.0060324.sg001.pdf]

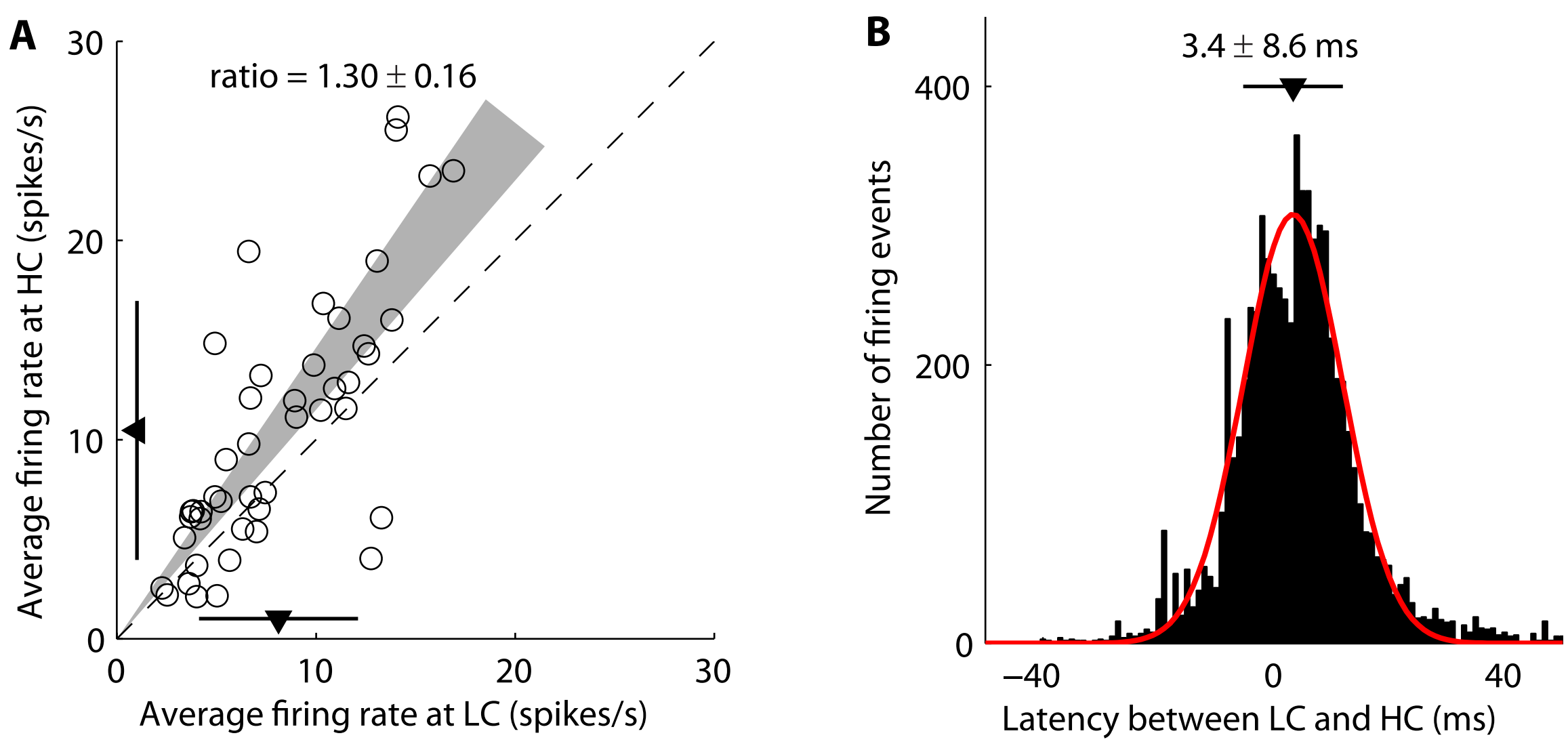

Figure S1. Distribution of Firing Rates and Latencies

(A) Distribution of average firing rates at HC vs. LC (n=45 cells). The dashed line is the unity line. The shaded area indicates the 95% confidence estimate of the mean HC/LC ratio of the average firing rates. The mean ± 1 standard deviation are represented along each axis by an arrowhead and bar.

(B) Distribution of latencies between LC and HC events, across all 7878 events from 45 cells. Latency is defined to be positive if LC occurs later than HC. The distribution of latencies has long tails and is only shown for the central ± 50 ms. The mean and standard deviation of the best-fitting Gaussian (in red) are indicated.
