# Supplementary material for: Timing Precision in Population Coding of Natural Scenes in the Early Visual System
Source: PLoS Biol. 2008 Dec 16;6(12):e324. doi: 10.1371/journal.pbio.0060324 (PMC2602720; doi:10.1371/journal.pbio.0060324)
Supplement: Supplementary file 3 [file pbio.0060324.sg002.pdf]

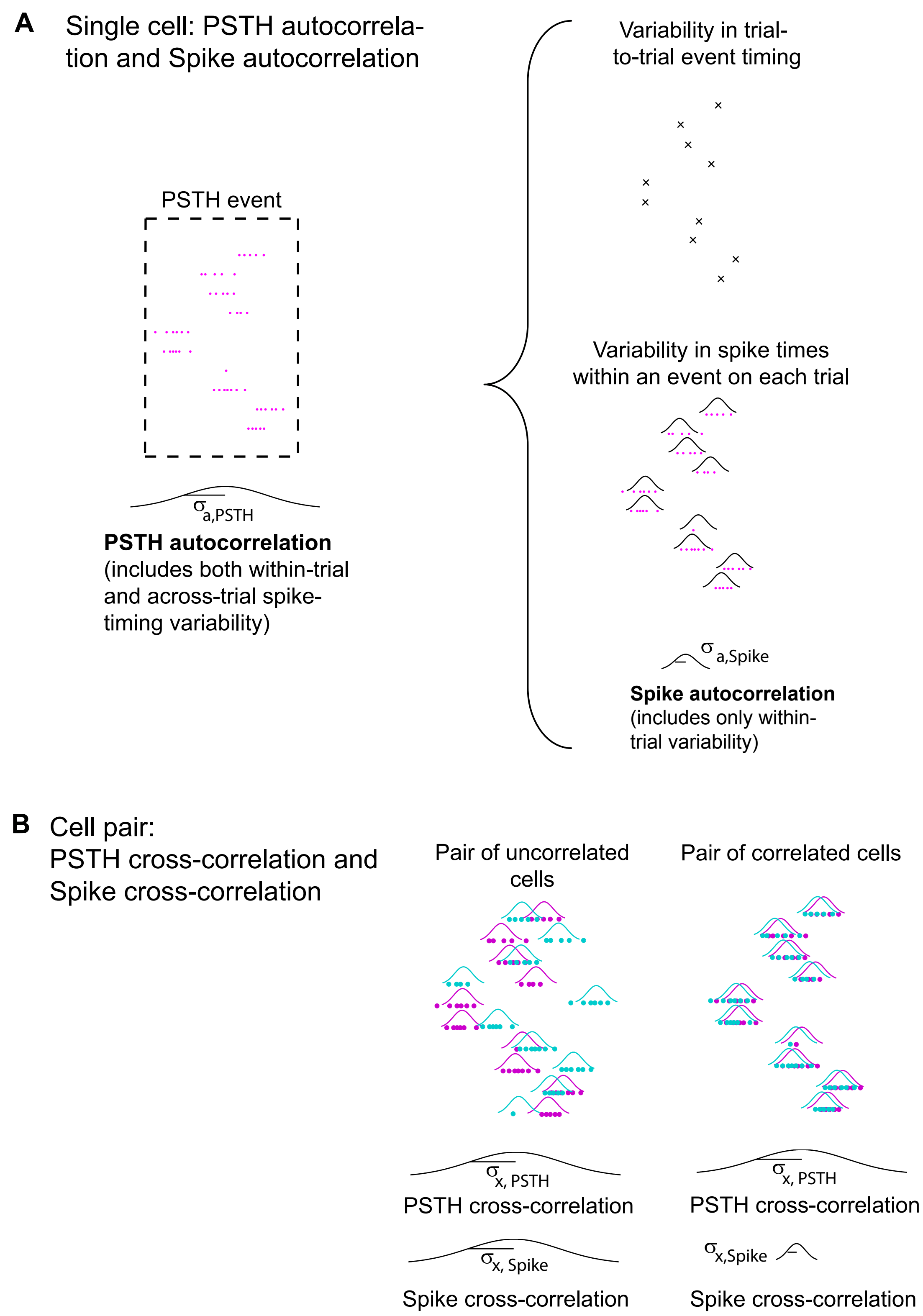

Figure S2. Connections between Spike Train Properties and Correlation Measures
