# Supplementary material for: Timing Precision in Population Coding of Natural Scenes in the Early Visual System
Source: PLoS Biol. 2008 Dec 16;6(12):e324. doi: 10.1371/journal.pbio.0060324 (PMC2602720; doi:10.1371/journal.pbio.0060324)
Supplement: Supplementary file 4 [file pbio.0060324.sg003.pdf]

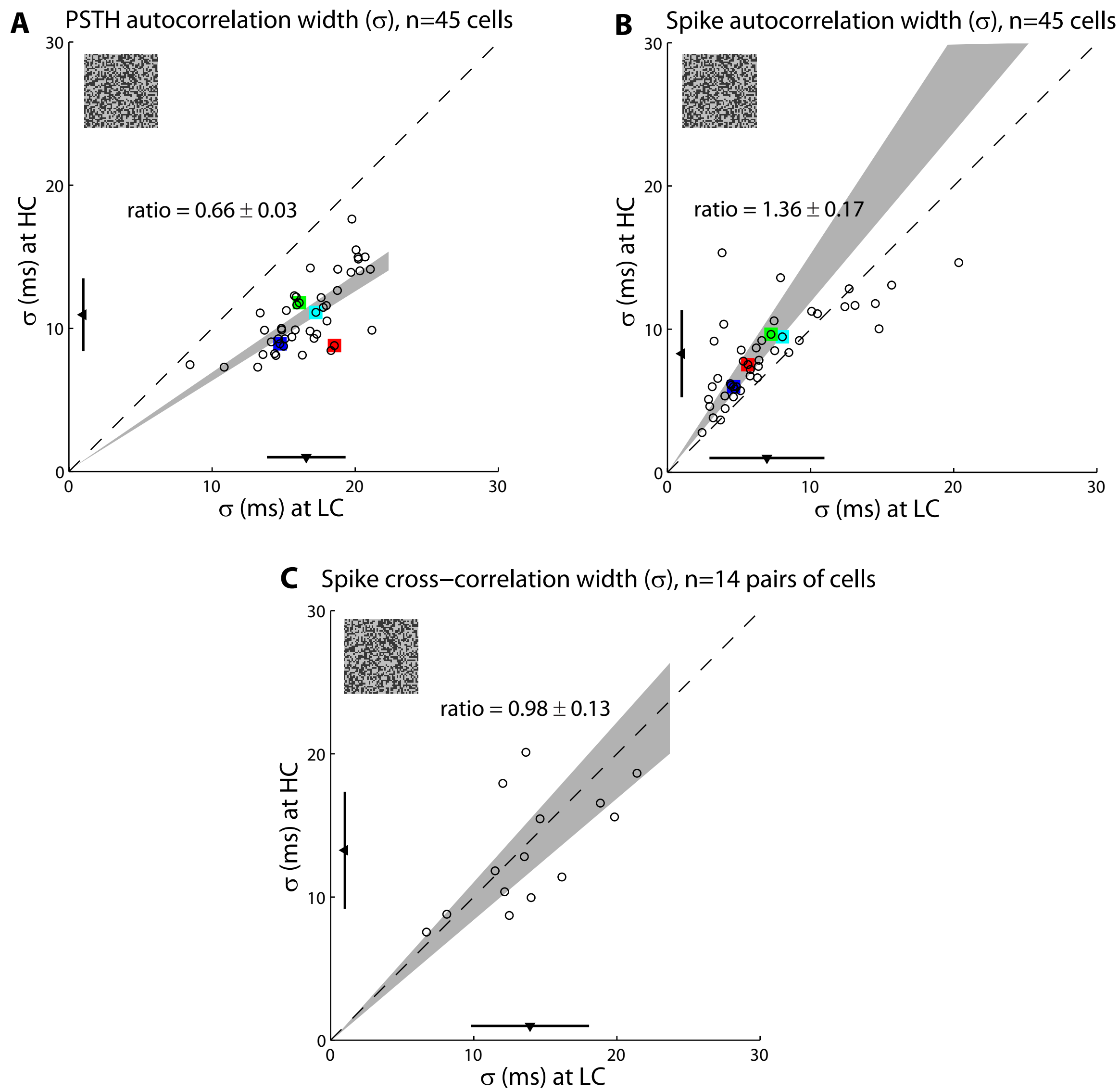

Figure S3. Results of Experiments in which Visual Stimulation Consisted of Spatiotemporal White Noise Stimuli

(A) Temporal width of the PSTH autocorrelation at HC vs. LC for the same 45 cells as in the main results. Same conventions as in Figure 2C in the main document.

(B) Temporal width of the spike autocorrelation at HC vs. LC for the same 45 cells. Same conventions as in Figure 2D.

(C) Temporal width of the spike cross-correlation at HC vs. LC for the subset of pairs that exhibited a measurable amount of cross-correlation with white noise stimuli. Same conventions as in Figure 4C.
