# Supplementary material for: Timing Precision in Population Coding of Natural Scenes in the Early Visual System
Source: PLoS Biol. 2008 Dec 16;6(12):e324. doi: 10.1371/journal.pbio.0060324 (PMC2602720; doi:10.1371/journal.pbio.0060324)
Supplement: Supplementary file 5 [file pbio.0060324.sg004.pdf]

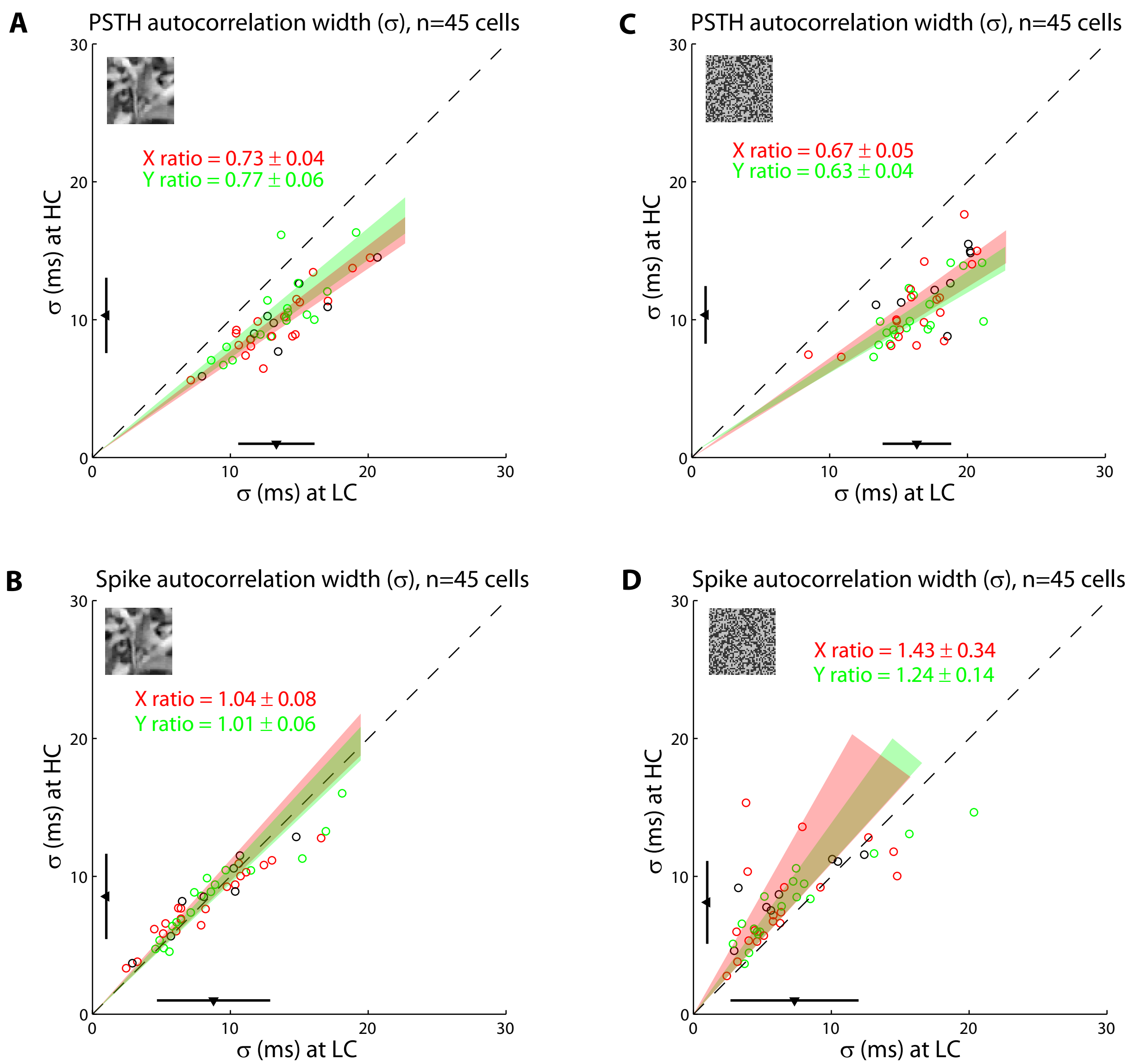

Figure S4. Control for X vs. Y Cell Type

The main single-cell results (see Fig. 2) hold for both X cells (red) and Y cells (green). Cells of unknown X or Y type are represented in black. The shaded areas correspond to the 95% confidence estimates of the mean HC/LC ratio of the autocorrelation widths, for X cells (red, n=19) and Y cells (green, n=18). Same conventions as in Figure 2 (C,D).

(A) Temporal width of PSTH autocorrelation for natural movies.

(B) Temporal width of spike autocorrelation for natural movies.

(C) Temporal width of PSTH autocorrelation for spatiotemporal white noise stimuli.

(D) Temporal width of spike autocorrelation for spatiotemporal white noise stimuli.
