# Supplementary material for: Timing Precision in Population Coding of Natural Scenes in the Early Visual System
Source: PLoS Biol. 2008 Dec 16;6(12):e324. doi: 10.1371/journal.pbio.0060324 (PMC2602720; doi:10.1371/journal.pbio.0060324)
Supplement: Supplementary file 6 [file pbio.0060324.sg005.pdf]

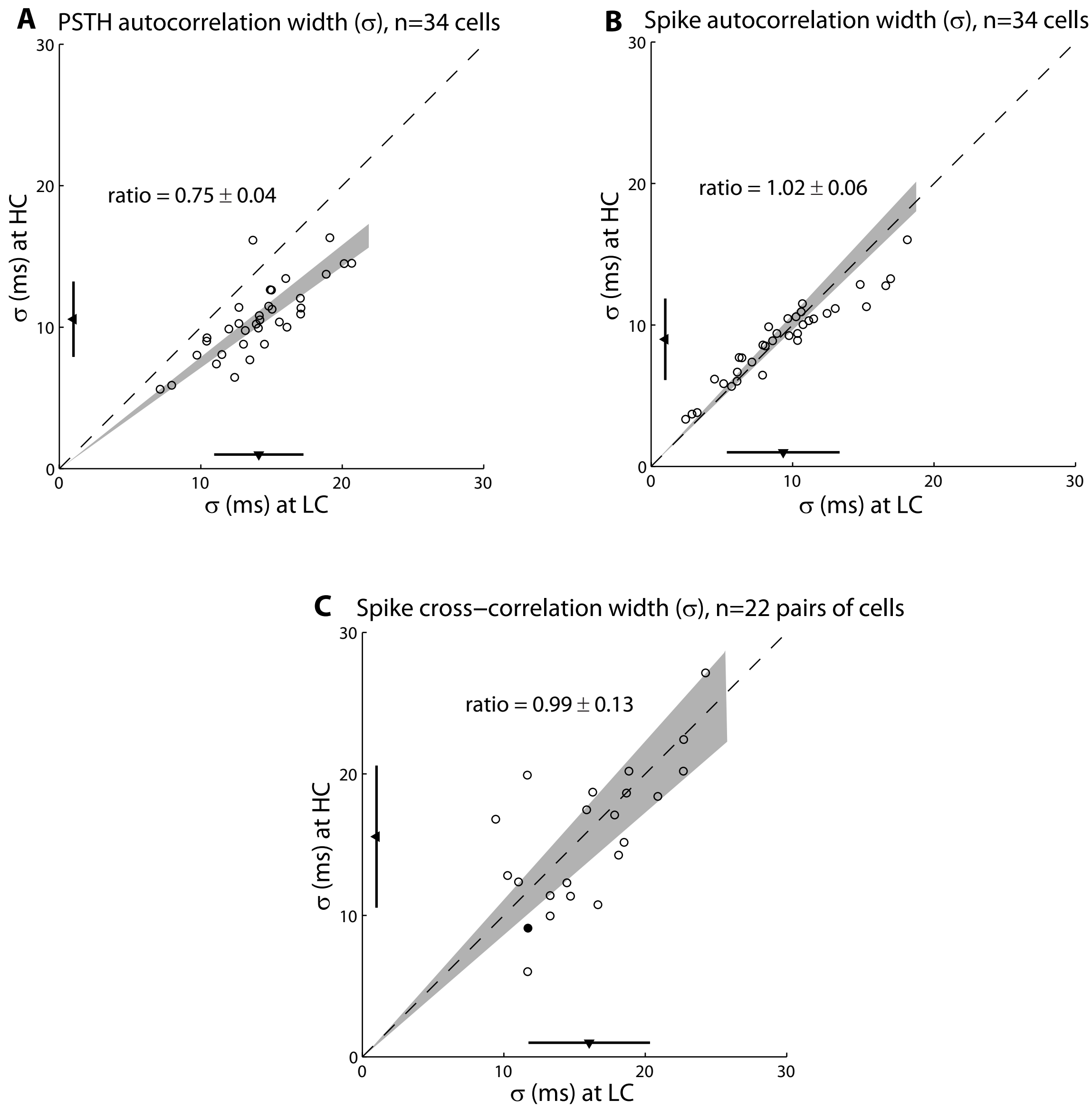

Figure S5. Control for the Occurrence of LGN Putative Calcium Bursts
(A) Temporal width of the PSTH autocorrelation at HC vs. LC (as in Figure 2C), for the 34 least bursty cells (out of 45 cells total).
(B) Temporal width of the spike autocorrelation at HC vs. LC (as in Figure 2D), for the same 34 least bursty cells.
(C) Temporal width of the spike cross-correlation at HC vs. LC, as in Figure 4C, for the 22 pairs (out of 41 pairs in the main analysis) involving the 34 least bursty cells from (A) and (B).
