# Supplementary material for: Timing Precision in Population Coding of Natural Scenes in the Early Visual System
Source: PLoS Biol. 2008 Dec 16;6(12):e324. doi: 10.1371/journal.pbio.0060324 (PMC2602720; doi:10.1371/journal.pbio.0060324)
Supplement: Supplementary file 7 [file pbio.0060324.sg006.pdf]

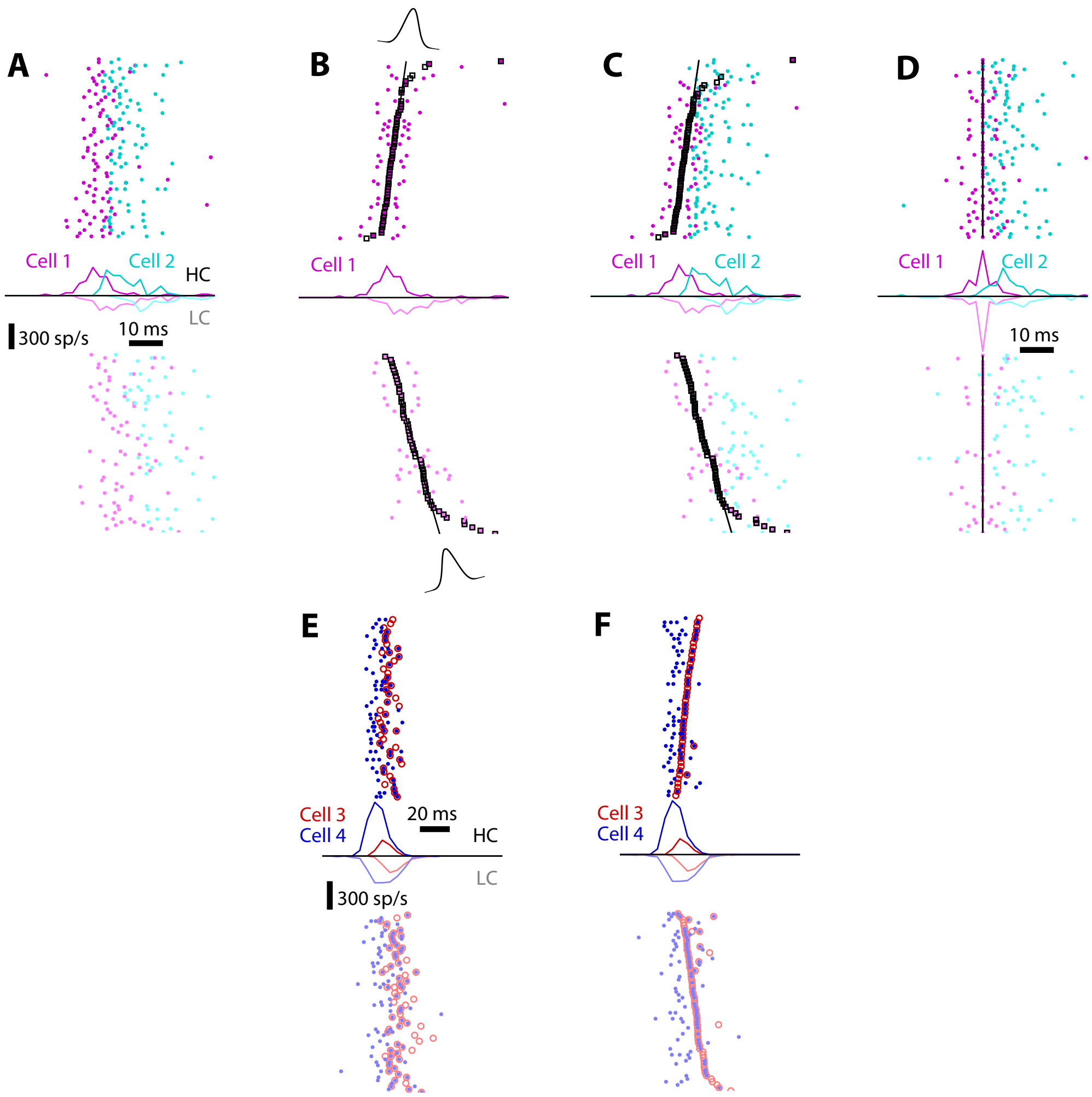

Figure S6. Examples of Raster Plots and PSTH for a Single Firing Event Shared by Two Cells

(A) Event shared by two X ON cells recorded simultaneously, showing raster plots and PSTHs. Only the repeats in which Cell 1 fired at least one spike are shown (56/64 repeats at LC, 63/64 at HC).

(B) Same event for Cell 1 only. The trials have been reordered based on the event time (calculated as the median spike time within the event for each trial, denoted with black circle; see Methods). The black lines are the best linear fit to the event times, excluding the ten repeats on which the cell fired the earliest and the ten repeats on which the cell fired the latest.

(C) Same data as in (A) where the trials are sorted as in (B) by increasing event time for Cell 1.

(D) Same data as in (A) where the trials are "dejittered" by aligning their median event time for Cell 1.

(E) Another event shared by two other cells from a different electrode penetration (Cell 3 spikes: red circles; Cell 4 spikes: small blue dots). These two cells, an X ON cell and a Y ON cell, share input from the same retinal afferent.

(F) Same as (E) except that the trials are sorted by increasing event time for Cell 3. Only the repeats in which Cell 3 fired at least one spike are shown (60/120 repeats at LC, 44/120 at HC).
