# Supplementary material for: Timing Precision in Population Coding of Natural Scenes in the Early Visual System
Source: PLoS Biol. 2008 Dec 16;6(12):e324. doi: 10.1371/journal.pbio.0060324 (PMC2602720; doi:10.1371/journal.pbio.0060324)
Supplement: Supplementary file 8 [file pbio.0060324.sg007.pdf]

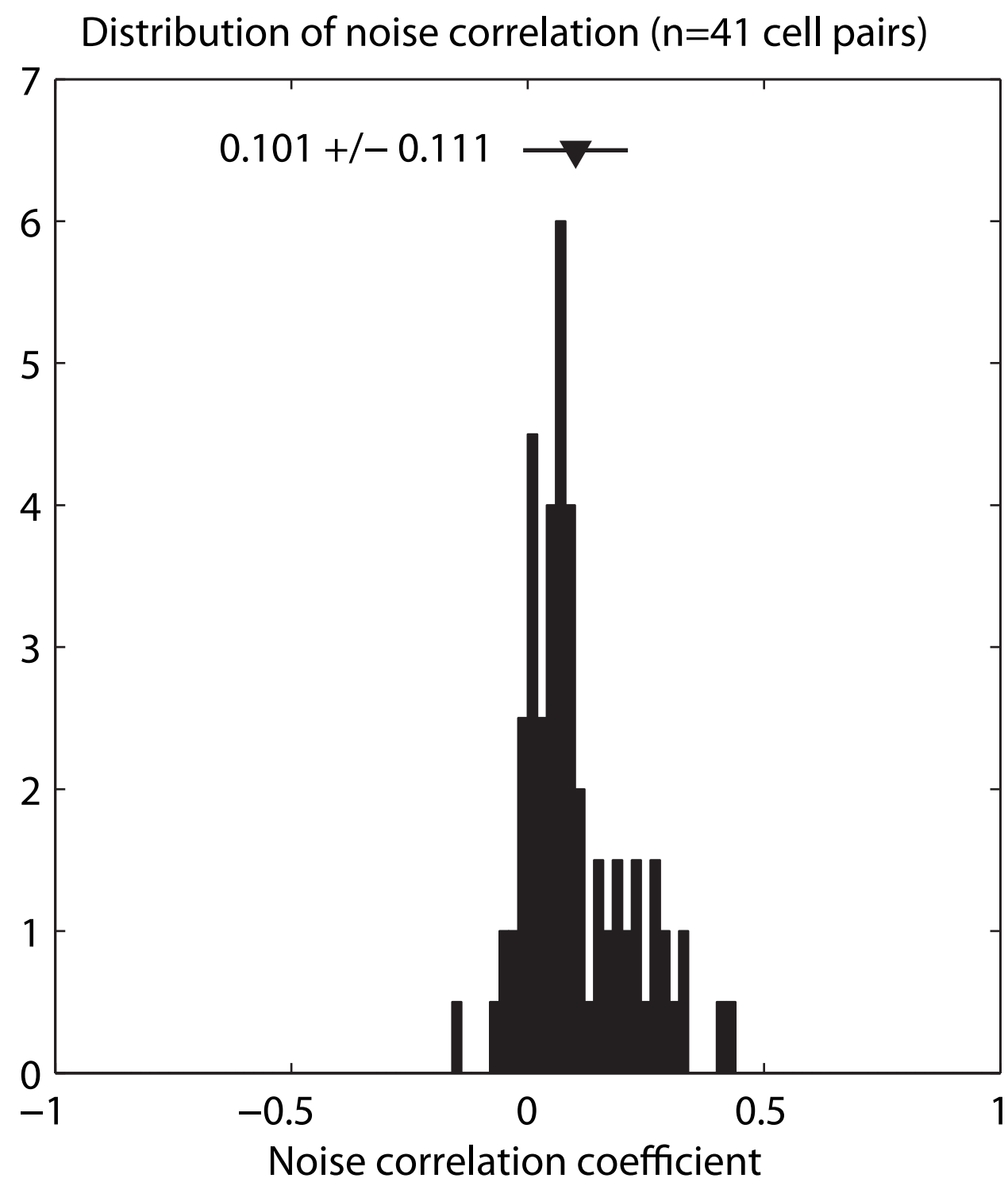

Figure S7. Distribution of Noise Correlation
The distribution is across all pairs included in the analysis. The mean and standard deviation are indicated.
