# Supplementary material for: Timing Precision in Population Coding of Natural Scenes in the Early Visual System
Source: PLoS Biol. 2008 Dec 16;6(12):e324. doi: 10.1371/journal.pbio.0060324 (PMC2602720; doi:10.1371/journal.pbio.0060324)
Supplement: Supplementary file 9 [file pbio.0060324.sg008.pdf]

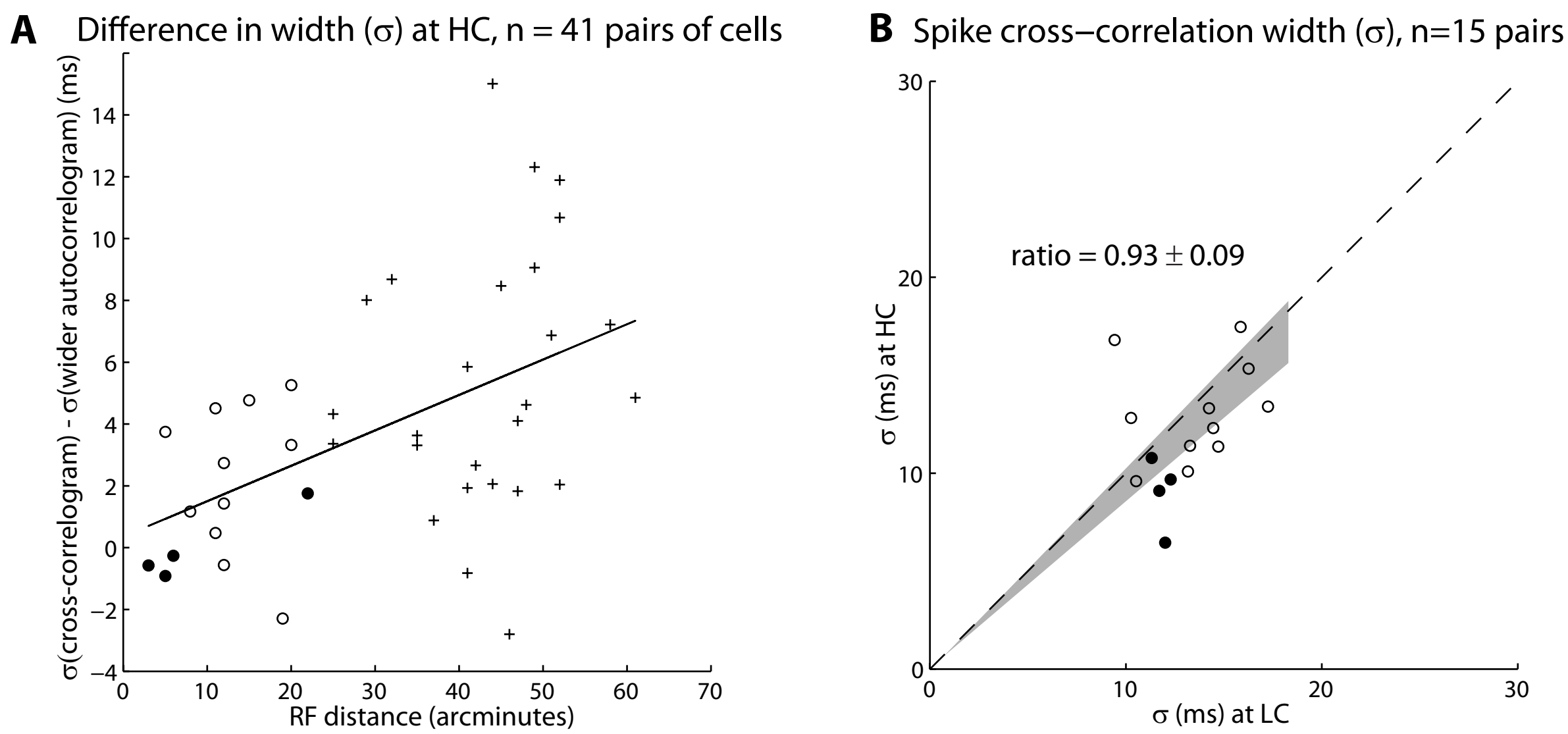

Figure S8. Control for Pairs of Cells with the Most Receptive Field Overlap, Which Are Likely to Fire at Similar Event Times

(A) Difference in width between the spike cross-correlation function and the wider of the two spike autocorrelation functions for each pair, as a function of the distance between the two receptive fields. Circles: 15 most closely-spaced pairs. The circles filled in black represent the four pairs of cells receiving common retinal input, as in Fig. 4C. Crosses: 26 remaining pairs.

(B) Temporal width of the spike cross-correlation at HC vs. LC for the 15 pairs shown by circles in (A) (n=15 pairs). Same conventions as in Fig. 4C.
